# What works for wellbeing? A systematic review of wellbeing outcomes for music and singing in adults

**DOI:** 10.1177/1757913917740391

**Published:** 2017-11-13

**Authors:** Norma Daykin, Louise Mansfield, Catherine Meads, Guy Julier, Alan Tomlinson, Annette Payne, Lily Grigsby Duffy, Jack Lane, Giorgia D’Innocenzo, Adele Burnett, Tess Kay, Paul Dolan, Stefano Testoni, Christina Victor

**Affiliations:** Centre for the Arts as Wellbeing, University of Winchester, Winchester SO22 4NR, UK; Doctor, Welfare, Health and Wellbeing, Institute for Environment, Health and Societies, Brunel University, London, Uxbridge, UK; Professor, Anglia Ruskin University, Cambridge, UK; Professor, College of Arts and Humanities, University of Brighton, Brighton, UK; Professor, College of Arts and Humanities, University of Brighton, Brighton, UK; Brunel University, London, Uxbridge, UK; Welfare, Health and Wellbeing, Institute for Environment, Health and Societies, Brunel University London, Uxbridge, UK; College of Arts and Humanities, University of Brighton, Brighton, UK; Welfare, Health and Wellbeing, Institute for Environment, Health and Societies, Brunel University London, Uxbridge, UK; Brunel University, London; Professor, Welfare, Health and Wellbeing, Institute for Environment, Health and Societies, Brunel University London, Uxbridge, UK; London School of Economics and Political Science, London, UK; London School of Economics and Political Science, London, UK; Professor, Ageing Studies, Institute for Environment Health and Societies, Brunel University London, Uxbridge, UK

**Keywords:** music, singing, systematic review, wellbeing, depression, older people

## Abstract

**Aims:**

The role of arts and music in supporting subjective wellbeing (SWB) is increasingly recognised. Robust evidence is needed to support policy and practice. This article reports on the first of four reviews of Culture, Sport and Wellbeing (CSW) commissioned by the Economic and Social Research Council (ESRC)-funded What Works Centre for Wellbeing (https://whatworkswellbeing.org/).

**Objective:**

To identify SWB outcomes for music and singing in adults.

**Methods:**

Comprehensive literature searches were conducted in PsychInfo, Medline, ERIC, Arts and Humanities, Social Science and Science Citation Indexes, Scopus, PILOTS and CINAHL databases. From 5,397 records identified, 61 relevant records were assessed using GRADE and CERQual schema.

**Results:**

A wide range of wellbeing measures was used, with no consistency in how SWB was measured across the studies. A wide range of activities was reported, most commonly music listening and regular group singing. Music has been associated with reduced anxiety in young adults, enhanced mood and purpose in adults and mental wellbeing, quality of life, self-awareness and coping in people with diagnosed health conditions. Music and singing have been shown to be effective in enhancing morale and reducing risk of depression in older people. Few studies address SWB in people with dementia. While there are a few studies of music with marginalised communities, participants in community choirs tend to be female, white and relatively well educated. Research challenges include recruiting participants with baseline wellbeing scores that are low enough to record any significant or noteworthy change following a music or singing intervention.

**Conclusions:**

There is reliable evidence for positive effects of music and singing on wellbeing in adults. There remains a need for research with sub-groups who are at greater risk of lower levels of wellbeing, and on the processes by which wellbeing outcomes are, or are not, achieved.

## Introduction

Policy makers have acknowledged the importance and complexity of subjective wellbeing (SWB).^1–4^ Since 2011, SWB (satisfaction with life, worthwhileness, happiness and anxiety) has been included in UK population surveys conducted by the Office of National Statistics (ONS). Links between cultural activities and wellbeing are acknowledged,^5^ with cultural engagement embedded in national-level data collection,^6^ and recognised in public health programmes.^7,8^ However, SWB is a complex conceptualisation of mental states that includes hedonic dimensions (both positive and negative feelings such as happiness, anxiety and stress) and eudaemonic dimensions (such as meaningfulness, purpose and worthwhileness).^9^ Increased understanding of the effects of cultural interventions on these dimensions of SWB is needed in order to inform policy and programme development.

The Economic and Social Research Council (ESRC)-funded What Works Centre for Wellbeing has commissioned evidence reviews in key areas, one of which is Culture, Sport and Wellbeing (CSW). Following consultation with stakeholders,^4^ the CSW review team identified four topics to be addressed between 2015 and 2018. The first topic, music in adults, is reported here. Music is a complex intervention encompassing diverse forms, including singing, music listening and playing instruments. It also includes different genres, such as choral music, rock and pop. An increasing body of evidence has examined health outcomes from music, particularly from singing.^10–12^ This review sought to examine a wide range of music interventions that might be linked with SWB rather than health and to differentiate which intervention types may be more closely linked with wellbeing.

## Methods

This systematic review followed the Preferred Reporting Items for Systematic Reviews and Meta-Analysis (PRISMA) guidelines.^13^ The review question was ‘What are the wellbeing outcomes of music and singing for adults and what are the processes by which wellbeing outcomes are achieved?’ The review anticipated that quantitative studies may report wellbeing outcomes, and these are reported here. Qualitative studies were included to illuminate the underlying processes by which wellbeing outcomes may be achieved; however, we decided to report the findings from the qualitative studies separately in order to be able to more fully examine the results from all the studies in the light of their respective methodologies.^14,15^

**Figure 1.**
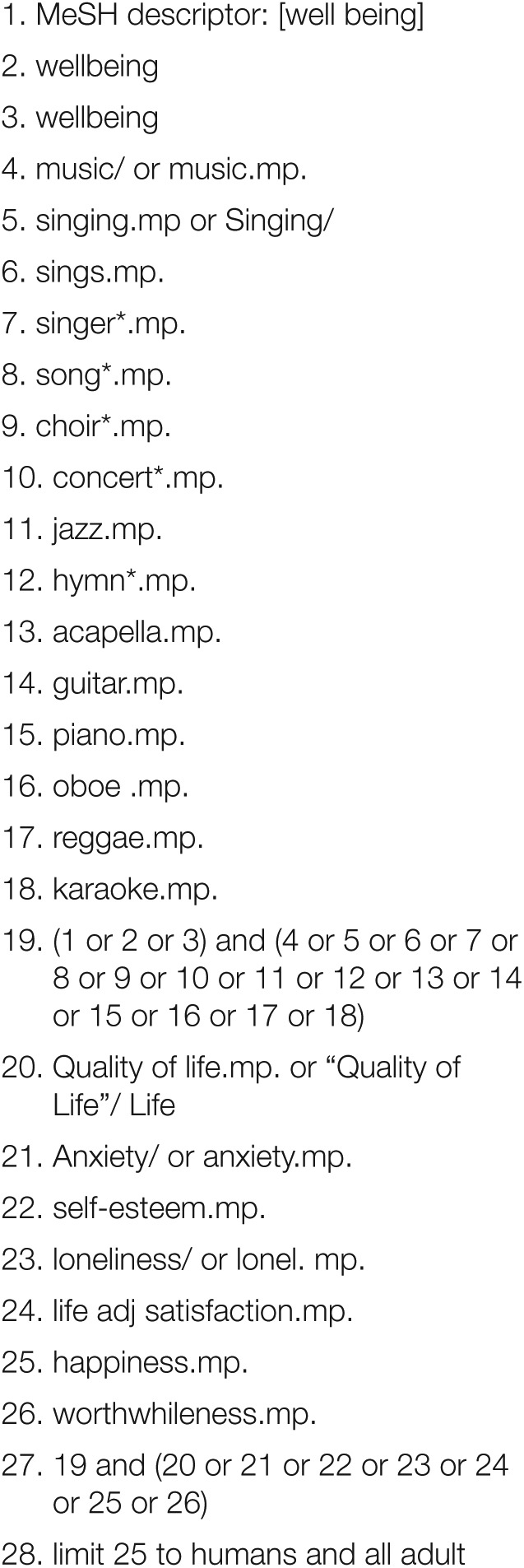
Search Strategy (Ovid MEDLINE)

The search strategy encompassed the period 1996–2016 and the following electronic databases: PsychInfo, Ovid MEDLINE, ERIC, Arts and Humanities Citation Index (Web of Science), Social Science Citation Index (Web of Science), Science Citation Index, Scopus, PILOTS and CINAHL. Search terms were developed in an iterative process involving a number of trial searches. Searches used a combination of controlled vocabulary (MeSH) and free-text terms (Figure 1). We also checked the reference lists of published papers to identify additional relevant articles. The protocol was registered on the PROSPERO International Prospective Register of Systematic Reviews (Registration number CRD42016038868).

Empirical studies in any language from countries economically similar to the United Kingdom were eligible if they assessed the relationship between individual or group music interventions and any recognised measure of SWB (not necessarily as the primary outcome) with adults. Regarding quantitative studies, we only included those with a concurrent comparator (CC). We excluded studies where the research subjects were paid professionals and where music was used for clinical purposes, such as pain management or symptom relief. However, music therapy interventions that sought to deliver wellbeing outcomes were included. Searching, screening and data extraction using standardised forms were undertaken by two reviewers, and discrepancies were resolved by deliberation, with a third researcher available for arbitration although this was not needed. GRADE and CERQual quality assessment schema were used to judge certainty/quality of evidence.^16^ A summary of the characteristics of included studies is provided in Table 1 (supplementary material).

Despite a great deal of heterogeneity across the studies, it was possible to undertake an exploratory meta-analysis on the effects of music interventions on anxiety and depression (see below).

## Results

**Figure 2:**
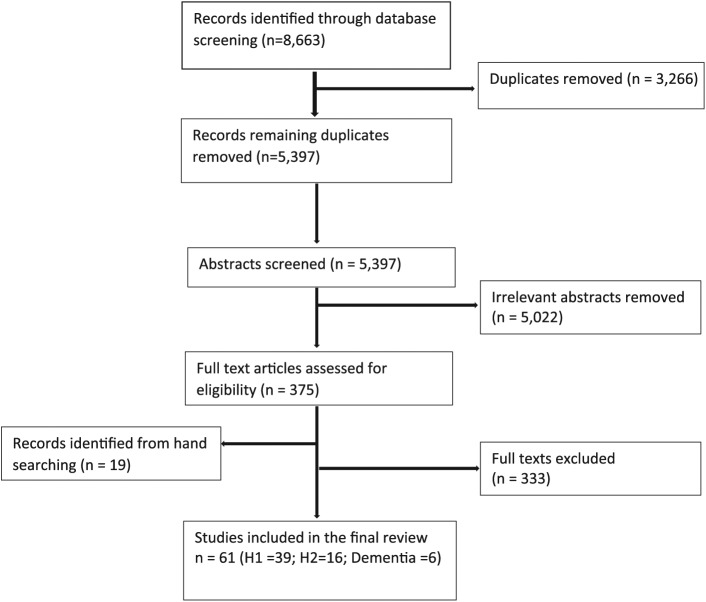
Literature search flow chart

The electronic searches returned 5,397 records (Figure 2), with 61 relevant studies remaining after abstract screening and full-text review. These studies fell broadly into studies with healthy (H1) populations (39) and 22 studies of participants with diagnosed conditions (H2), including six studies of SWB in people with dementia.

### Characteristics of quantitative studies included in the review

Study characteristics of quantitative studies, including study size, participants, wellbeing measures, outcomes and quality assessment are described in Table 1 (supplementary material). Many wellbeing measures were used, including anxiety, depression, mood and quality of life. While there was no consistency in how wellbeing was measured across the studies, there was a greater emphasis on negative dimensions than on positive dimensions of wellbeing.

Many activities were reported, most commonly music listening and regular group singing. The data show that some projects, such as community choirs, seem more likely to be attended by women than men. Where ethnic backgrounds and other demographic characteristics of participants were recorded, most participants were white and relatively well educated. However, several interventions were aimed specifically at other groups including males, marginalised groups including migrants and people in justice settings.

The review includes a wide range of study designs from many countries. Sample sizes for quantitative studies ranged from nine to 750. Risk of bias may have arisen from methodological challenges including, in quantitative studies, recruitment, randomisation, small sample sizes, attrition and prevalence of single-site studies in specific contexts.

## Discussion

### Discussion of quantitative findings

Given the heterogeneity of studies, we have concentrated on different population groups across the adult life span including students, targeted adult populations, and older adults.

#### Students

Ten quantitative studies in educational settings mostly examined brief (5–10 min) music listening interventions, often with a focus, such as listening to music during exercise (see Table 1, supplementary material). There were several randomised control trials (RCTs) but these tended to be small, single studies with less than 20 participants allocated to each group. A study of mostly male PE students reported reduced scores on the State-Trait Anxiety Inventory following listening to music during a brief treadmill running test,^19^ and a non-random study reported improvements in anxiety but not in enjoyment for nine male PE students undertaking a similar intervention.^17^ A study of mostly female students compared four small group conditions and reported improved mood and reduced negative affect for music and dancing, although cycling and sitting had no reported effects.^25^ Another study of male and female students compared four music listening, art and control conditions, reporting improved scores on the STAI-S and the Profile of Mood States (POMS) following music listening.^23^

A longer (30 min) listening activity was examined in a study comparing four conditions, reporting higher relaxation scores for own choice and classical music compared with hard rock and silence.^24^ In this study, anxiety scores for all groups except for the hard rock group significantly decreased. A longer study of 80 male students compared listening to slow-paced Indian instrumental music with silence for 30 min daily for 20 days, reporting a significant reduction of 2.7 points on the STAI for the intervention group as well as improvements in mood, while no significant changes were reported for the control group.^40^

Few of these H1 studies examined interventions other than music listening. However, one non-random study of students compared 30-min sessions of solo singing, group singing and swimming, reporting improvements in mood for all student groups, with the strongest changes reported for swimming.^52^ A musical presentation (MP) activity, in which participants in a group present themselves using music of their choice, was linked with enhanced sense of purpose and self-consciousness in a quasi-experimental study of female students.^22^

Only one study in this group examined playing musical instruments. This RCT of 154 non-musicians that compared a brief session of playing percussion to joyful music with simulated playing to computer-generated tones reported significant improvements in elements of the POMS, with depression, anxiety and fatigue decreasing in the music group but not in the control group. The music group also showed increased vigour, which decreased in the control group, while irritability increased in the control group but not in the music group.^43^

In addition to these studies of healthy student volunteers, two H2 studies examined the effects of music interventions for students with diagnosed depression. A study of 80 nursing students comparing a 10-week programme of listening to recorded Chinese music with usual activity reported significant improvements in depression.^31^ A small study of male and female students compared a 10-week music therapy programme with no music therapy, reporting improvements in anxiety and depression in the music group compared with controls.^55^

#### General adult populations

Nine studies assessed music in healthy general adult populations, including four studies in prisons. Five of the nine assessed brief listening interventions. A repeat-measure study of 100 hospital employees compared a music listening session with music and visual imagery, massage therapy and social support, reporting improvements on STAI and POMS for all groups.^37^ A comparison of daily music listening over 18 days with mindfulness meditation in a study of 56 adult employees reports no significant reductions in stress, although the stress in the waiting list control group increased.^26^ Two larger studies of pregnant women compared listening to relaxing music for 30 min a day for two weeks with usual activity. In both, music was associated with significant reductions in stress, anxiety and depression compared with the control condition.^29,30^ A quasi-experimental study of adult prisoners compared three weeks of listening to relaxing background music with no exposure, reporting reduced anxiety and improvements in anger after music listening.^21^

Studies with offender populations addressed music making as well as music listening. A non-random study of young offenders compared a 10-week programme of music making, including songwriting, playing and performing, with art or educational activities, reporting increased self-esteem in the music and education groups and improvements in emotional state for music and arts.^18^ A study of music therapy in which prisoners played instruments, sang and recorded music reported significant improvements in anxiety in the music group after two weeks; however, no data are available for the control group.^38^

There were two studies of choirs, one of which was a small non-random study with adult prisoners that compared nine weeks of group singing leading to a performance with usual activity, reporting no differences between groups in overall wellbeing scores, although participants in a choir involving volunteers from the community showed improvements in sociability, joviality, emotional stability and happiness compared with controls.^33^ Another open-access community-based choir study with healthy volunteers reported an increase in positive feelings after seven weeks of group singing but not after a comparator chatting activity, while negative feelings decreased significantly after singing but not after chatting.^44^

#### Healthy older adults

Seven studies with healthy adults were divided between music listening (3), singing (3) and music making (1). Three community-based studies of music listening in healthy older adults indicate an association between music listening and wellbeing. Two studies compared listening to music using headphones for 30 min a week for four weeks, reporting significant improvements in quality of life compared with controls^45^ and a 2-point reduction in mean Geriatric Depression Scale (GDS) scores that was significant compared with controls.^27^ In another study, a difference of 2.79 points in GDS scores between intervention and control groups was reported after eight weeks of music listening.^28^

Wellbeing outcomes were identified in two larger studies of community singing for mostly female healthy older adults. One study of 258 participants over five sites compared a 14-week singing programme with usual activities, reporting significant differences between the groups on the York SF 12 mental health–related quality-of-life scale, and the Hospital Anxiety and Depression (HAD) scale after three months, with significant differences in mental health–related quality of life in favour of group singing after six months.^36^ This research built on an earlier study of 166 participants that compared a 30-week choral singing project with usual activity. This showed significant differences after 12 months in morale, depression and loneliness for intervention groups compared with controls. While both groups evidenced a decline in morale and loneliness, this was slighter for the comparison group who showed a reduced risk of depression after 12 months.^32^ In contrast, a non-random community study evaluating community singing, music appreciation classes and music therapy for older adults over one academic year reported no significant wellbeing outcomes using an ad hoc questionnaire.^49^ Only one study of older adults examined playing musical instruments, reporting improvements in wellbeing both in older adults taking music lessons and those not taking lessons after 10 weeks.^47^

#### Music for targeted health populations

Eight quantitative studies examined music that was targeted at people with diagnosed health conditions. A non-random comparison of singing with usual care in 113 adults with a range of chronic conditions found that singing was associated with improvements in quality of life and positive affect.^50^ A study of patients with chronic obstructive pulmonary disease (COPD) compared weekly singing with attending a film club for eight weeks, reporting mental wellbeing improvements for both groups.^46^ Another study reported small improvements in anxiety, and significant improvements in depression, for a 4-week music therapy group for stroke patients compared with usual activity.^42^ Two small studies in palliative care settings examined brief (30 min) music therapy sessions, reporting improvements in wellbeing for a music therapy protocol compared with relaxation sessions,^53^ and in spirituality compared with no music therapy.^54^

The findings from studies of healthy older adults were not necessarily replicated in studies of adults with diagnosed health conditions or risk factors. For example, a study comparing the effects of music listening and relaxation on anxiety for older people with hypertension in a residential care setting reported no significant changes following 28 days of daily activity.^20^ Furthermore, a small study comparing twice weekly 30-min sessions of secular singing, religious singing and story-based reminiscence in a residential care setting reported no significant wellbeing outcomes after six weeks.^41^ A case-controlled study compared 15 months of tai chi, playing a musical instrument or singing in adults aged 51–85 years with risk factors for chronic disease, reporting improvements in resilience and depression for all intervention groups compared with controls, with the lowest depression rates for tai chi and dancing groups.^51^

#### Music and dementia

Challenges of researching SWB in people with dementia were apparent, and studies that measured SWB by proxy were excluded, leaving only three quantitative studies of music and wellbeing in dementia care that included a CC. Furthermore, a lack of specificity regarding diagnosis, and a tendency to include participants with relatively high baseline scores, makes it difficult to interpret these studies.^56^ One study compared group singing, listening and playing sessions with an interactive reading group, reporting no significant changes on the anxiety or on the Dementia Quality of Life Scale or the Geriatric Depression Scale.^34,35^ A five-centre study of 89 participants with dementia and their carers showed increased quality-of-life scores for a music listening group compared with usual activity, although the differences between the groups had levelled off by nine months.^48^ Evaluation of a 24-week individual music therapy intervention with 30 nursing home residents reported a significant reduction in anxiety and a 7.8 reduction in GDS scores, with no significant change reported for the control group; the differences in depression between the two groups were persistent at eight weeks post intervention.^39^

### Exploratory meta-analysis

**Figure 3:**
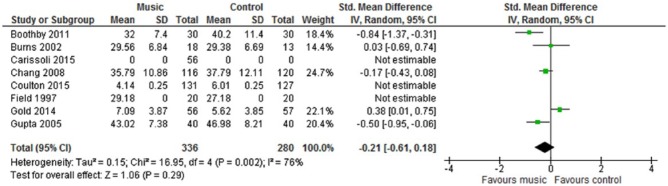
Forest plot of anxiety outcome results

**Figure 4:**
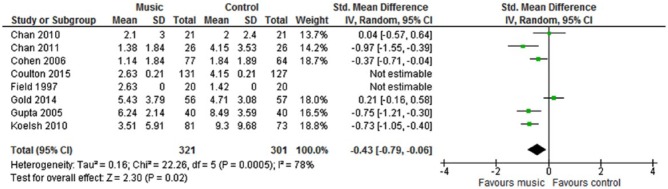
Forest plot of depression outcome results

We tabulated characteristics and results of all included studies. Five studies contributed to the meta-analysis on anxiety and six studies contributed to the meta-analysis on depression (Figures 3 and 4). All outcomes were continuous measures. When standard errors, ranges or 95% confidence intervals were provided, standard deviations were calculated using standard formulae. Where no measure of spread was given, the study was still entered. We used Review Manager (version 5.3.5, Cochrane Library) for the meta-analyses. We used random-effects models because of heterogeneity of participants and interventions, although this approach only partly removes effects of heterogeneity.^57^ A variety of anxiety and depression outcome measurement scales were used in the studies, so we employed standardised mean differences (SMD) as the meta-analysis metric. There were insufficient studies reporting the same outcome to warrant risk of publication bias assessment by use of funnel plots.

The analysis showed that music had no statistically significant effect on anxiety (SMD −.21 (95% CI −.61 to +.18) but improved depression at follow up (–.43 (95% CI −.79 to −.06). Heterogeneity was high for both anxiety and depression, with *I*^2^ varying between 76% and 78%.

### Quality of the evidence

Methodological strengths were noted in some areas, particularly in relation to research with older people. Common methodological limitations leading to risk of bias were identified (Table 1, supplementary material). There was a preponderance of small, single-site studies. Sampling issues and limitations regarding allocation to intervention and control groups were identified in several quantitative studies, some of which examined brief interventions and did not include longer term assessment. Longer term programmes reported risk of contamination affecting the control group or difficulties in controlling for confounding variables such as music listening practices, exercise or daily activities. In some studies, baseline conditions may not have been sufficiently pronounced to show a change in response to music. Nevertheless, approximately two-fifths of the evidence overall was graded moderate quality, with approximately one-sixth of studies graded high quality.

## Limitations

The large number of hits following initial searches and the overlap between clinical and wellbeing interventions means that it is possible that some relevant evidence was not included in the review. However, our comprehensive search strategy, the pre-publication of our protocol on PROSPERO, dual screening and data extraction and independent quality assessment using GRADE and CERQual criteria ensured a rigorous process. Taking published studies as the sole evidence increases the potential risk of publication lag. However, a separate grey literature review included data from unpublished studies completed in the last three years.^58^

## Conclusion

This article has discussed 37 quantitative studies of SWB outcomes for music and singing across the life course. Overall, music listening interventions seem to be the most frequently evaluated intervention, although group singing has also been the focus of a number of studies. Few studies have examined the effects of playing musical instruments, and further research is warranted in this area. Given the heterogeneity of studies, the diversity of interventions and the different intervention durations, it is difficult to generalise from them. A further difficulty is the overlap between clinical and non-clinical research, with some interventions that are described as music therapy seeming to have similar attributes to those that are not described in this way. Nevertheless, our exploratory meta-analysis suggests a positive association between music and improved depression.

Taken together, the studies broadly support the use of music and singing to enhance wellbeing and reduce or prevent depression in adults across the life span. For older adults, there is convincing evidence that regular participation in community music and singing activities can enhance and maintain wellbeing and prevent isolation, depression and mental ill health. There is also some evidence that targeted music and singing interventions can contribute to improved mood and reduced anxiety in specific groups including young adults, pregnant women and prisoners. Furthermore, interventions such as group singing may lead to improvements in wellbeing and quality of life for adults with a range of chronic conditions and in sensitive settings such as palliative care.

A key challenge may be recruiting from sub-groups who are at greater risk of lower levels of wellbeing. Future research may need to target participants with baseline wellbeing scores that are low enough to record any significant or noteworthy change following a music or singing intervention. There seems to be a tendency towards recruiting participants to community choirs who are female, white and relatively well educated. This is reinforced by supporting analysis of longitudinal data suggesting that engagement in music is positively correlated with younger age, White ethnicity and higher education level.^9^ Further research is needed to explore nuanced responses and to identify the individual, interpersonal and social factors, including gender, ethnicity and socioeconomic status that may mediate wellbeing outcomes in specific contexts. Addressing issues of context, social diversity and wellbeing inequalities represents an important focus point for policy, practice and research agendas on music singing and wellbeing.

## Supplementary Material

Supplementary material
