# Supplementary material for: What works for wellbeing? A systematic review of wellbeing outcomes for music and singing in adults
Source: Perspect Public Health. 2017 Nov 13;138(1):39–46. doi: 10.1177/1757913917740391 (PMC5753835; doi:10.1177/1757913917740391)
Supplement: Supplementary material [file PPH740391_Supplementry_REV1.pdf]

Table 1

Characteristics of included quantitative studies.

Authors/title/DOI	Participants recruited (completed)	Wellbeing measures	Wellbeing outcomes reported	Interven-tion and duration	Study design	Quality/bias
Aloui et al.[17]/https://doi.org/10.1371/journal.pone.0136400	9 male PE students aged 21 ± 1.1 years	7-item subscale of the Enjoyment Inventory. Pre- and post assessment	Improvements in cognitive anxiety<br>No changes in enjoyment or self-confidence	10 min	Non-random assessment of exercise with or without music	Very small, non-random sample. Single-site study. Short intervention time.
Anderson and Overy.[18]/http://dx.doi.org/10.1386/ijcm.3.1.47/1	19 (14) male offenders aged 17–21 years.	The Rosenberg Self-Esteem Scale.<br>The Emotion Scale.<br>Baseline assessment and at end.	Increased self-esteem in music and education groups. Emotion scores decreased in the music and arts groups and increased in the education group.	10 × 3.5 h sessions	Non-random assessment of music, art or education. Music sessions involved preparing and recording songs using guitar and vocals.	Small, non-random sample. Single-site study. No account of missing data.
Baldari et al.[19]/PMID 20842088/https://ncbi.nlm.nih.gov/pubmed/20842088	30 (26) male and female PE students aged 20–28 years	State–Trait Anxiety Questionnaire (STAI-S).<br>Pre- and post assessment	Reduced anxiety in the intervention group, from $M = 39.7$ (±1.8) to 32.1 (±2.0) compared with 35.2 (±2.5) to 33.3 (±2.2) for controls	Brief treadmill running test	RCT comparing listening to music with no music while exercising	Small sample. Short intervention time. Single-site study
Bekiroğlu et al.[20]/http://dx.doi.org/10.1016/j.ctim.2013.03.005	60 older adults with hypertension living in a residential facility.	Hamilton Anxiety Scale (HAM-A)<br>Baseline assessment and at end	No statistically significant change in values	28 days	Parallel group, RCT comparing daily music listening with relaxation	Single-site study. Baseline anxiety levels may not be sufficiently high enough to show a change in response to music<br>Control was to rest and sit for 25 min/day × 28 which may not be a good comparison.

(Continued)

Table 1 (Continued)

Characteristics of included quantitative studies.

Authors/title/DOI	Participants recruited (completed)	Wellbeing measures	Wellbeing outcomes reported	Interven-tion and duration	Study design	Quality/bias
Bensimon et al.[21]/ http://dx.doi.org/10.1177/0306624X13511587	48 adult male prisoners.	STAI.<br>State–Trait Anger Expression Inventory (STAXI). Assessment at baseline, during exposure and at the end	After 1 week, mean anxiety reduced significantly ($p = .002$) in the music listening group but not for the comparison group. After 3 weeks, a substantial and significant change was maintained and ($p = .001$). Anger scores improved moderately in the intervention group, and at 3 weeks, the difference was significant ($p = .047$)	3 × 45-min sessions of background music for 6 days a week for 3 weeks	Quasi-experimental study comparing exposure to relaxing background music on the wing with no exposure.	Small sample, immediate effects measured, no longer term assessment of effects.
Bensimon and Gilboa.[22]/ http://dx.doi.org/10.1016/j.aip.2010.03.002	26 female criminology students and 26 male and female drug users from a rehabilitation programme for ex-offenders	Purpose in Life test (PIL)<br>Self-Consciousness Scale (SCS). Assessment at the beginning and a week after the last MP session	Students ($n = 11$) in the intervention group showed significant ($p < .01$) increases in PIL and changes SCD ($p < .001$). Drug users ($n = 13$) in the intervention group showed improvements in PIL ($p < .01$) but not in SCS. No changes in PIL or SCS were found for the controls (15 students, 13 drug users)	7× weekly sessions	Quasi-experimental study comparing Musical Presentation (MP) – a therapeutic activity in which participants in a group present themselves using music of their choice - with usual activities	Small sample. Ages not specified, gender asymmetry in the groups. Non-random assignment. Comparator condition underspecified. Lack of follow-up assessment
Boothby and Robbins.[23]/ http://dx.doi.org/10.1080/07421656.2015.994428	60 male and female university students (mean age = 21.5 years)	Profile of Mood States (POMS)<br>STAI-S<br>Pre- and post assessment	In the music listening conditions, both measures improved significantly after the intervention ($p < .01$)	10 min	RCT comparing four music listening and art conditions with no music/art	Small sample, single-site study, short intervention time

(Continued)

Table 1 (Continued)

Characteristics of included quantitative studies.

Authors/title/DOI	Participants recruited (completed)	Wellbeing measures	Wellbeing outcomes reported	Intervention and duration	Study design	Quality/bias
Burns et al.[24]/https://doi.org/10.1093/jmt/39.2.101	60 male and female university students (mean age = 21.6 years)	STAI<br>Relaxation ratings.<br>Pre- and post assessment	For the intervention group, relaxation scores were significantly higher for the own choice music and classical music groups after listening to music. STAI scores decreased for all groups after listening to music. The control, self-selected music, and classical music groups' state anxiety scores all significantly decreased compared to the hard rock group	30 min	RCT comparing four conditions: listening to classical, rock, own choice music or silence	Convenience sample of predominantly Caucasian college students. Small groups, short intervention time
Campion and Levita.[25]/http://dx.doi.org/10.1080/17439760.2013.848376	60 male and female ($n = 51$) undergraduate students (mean age 20.4 years)	Subjective Exercise Experiences Scale (SEES).<br>Pre- and post assessment	SEES scores in the music group ($n = 14$) increased significantly positive ($p = .003$) while negative affect was significantly reduced ($p = .011$). Dancing also significantly increased measures of positive affect although cycling and sitting had no effect	5 min	RCT of four conditions: listening to music, dancing, cycling or sitting quietly	Small groups, may not be generalisable beyond predominantly young female sample, short intervention time
Carissoli et al.[26] /http://dx.doi.org/10.1089/cyber.2014.0062	56 employees	Mesure du Stress Psychologique (MSP).<br>Baseline assessment and at end.	No significant differences were reported although the waiting list control group showed a constant increase in stress on all MSP dimensions	2 × 15 min sessions a day for 18 days	RCT comparing listening to suggested music while doing nothing else with mindfulness mediation	Small sample. Risk of contamination
Chan et al.[27] https://doi.org/10.1016/j.ctim.2010.02.004	42 older males and females at a community services centre	Geriatric Depression Score (GDS-15). Assessment every week for 4 weeks	After 4 weeks, the music group showed a 2 point reduction in mean GDS scores that was statistically significant (.001). No reduction in depression was reported for the control group	30 min a week for 4 weeks	RCT comparing music listening using headphones with no music	Small sample. Possible Hawthorne effect. Some participants did not like using headphones and this may have influenced the results

(Continued)

Table 1 (Continued)

Characteristics of included quantitative studies.

Authors/title/DOI	Participants recruited (completed)	Wellbeing measures	Wellbeing outcomes reported	Interven-tion and duration	Study design	Quality/bias
Chan et al.[28]/ http://onlinelibrary.wiley.com/doi/10.1111/j.1365-2702.2011.03954.x/abstract	52 male and female adults aged over 55 years	GDS Assessment weekly for 8 weeks	After 8 weeks, the music group showed a difference of 2.79 points in GDS scores that was significant ($p = .016$). No significant difference was reported for the control group	30 min a week for 8 weeks	RCT comparing listening to own choice relaxing music with no music	Small sample. Snowball sampling from researcher network was used in recruitment although participants were randomised once recruited. Possible Hawthorne effect. Risk of contamination in the control group and possible confounding effects of music listening practices in intervention group
Chang et al.[29]/ https://doi.org/10.1016/j.ctim.2015.05.002	241 (236) Pregnant women expected to have uncomplicated vaginal deliveries	Pregnancy Stress Rating Scale (PSRS), Perceived Stress Scale (PSS)<br>Edinburgh Post Natal Depression Scale (EPDS). Baseline assessment and at end	After 2 weeks, women in the music group had significantly lower scores than the control group for PSS, STAI and EPDS	30 min a day for 2 weeks	RCT comparing listening to relaxing music with usual care	Study could not differentiate between different types of music or consider usual music listening habits. Short intervention time
Chang et al.[30]/doi: 10.1111/j.1365-2702.2007.02064.x	320 (296) pregnant women expected to have an uncomplicated vaginal delivery	PSRS<br>PSS<br>Maternal-Fetal Attachment Scale (MFAS)<br>Baseline assessment and at end	Significant differences between pre-test and post-test PSRS scores but not for PSS scores between experimental group and the control group	30 min a day for 2 weeks	RCT comparing music listening with usual activity	Study could not consider the effects of daily activities. The effects of self-selected music versus investigator-selected music were not compared. Short intervention time

(Continued)

Table 1 (Continued)

Characteristics of included quantitative studies.

Authors/title/DOI	Participants recruited (completed)	Wellbeing measures	Wellbeing outcomes reported	Interven-tion and duration	Study design	Quality/bias
Chen et al.[31]/http://dx.doi.org/10.1111/ijn.12236	80 (71) nursing students aged 16–20 years with Depression Mood Self-Report Inventory for Adolescence (DMSRIA) scores of 8 points or higher	Depression Mood Self-Report Inventory for Adolescence (DMSRIA)<br>Baseline assessment and at end	The intervention group showed a significant ($p = .001$) reduction in mean scores of from pre-mean of 15.52 (SD = 1.59) to post mean of 9.82 (SD = 3.87). Control group mean DMSRIA scores were pre 15.03 (SD = 1.88) post 15.53 (SD = 2.08)	40 min of listening to recorded Chinese music every week for 10 weeks	RCT comparing music listening with usual activity	Single-site study: limited generalisability. Short intervention time
Cohen et al.[32]/https://dx.doi.org/10.1093/geront/46.6.726	166 male and female older adults (mean age = 79 years) in a community setting. 79% were females	Philadelphia Geriatric Center<br>Morale Scale Loneliness Scale-III<br>GDS–Short Form<br>Assessment at baseline and after 12 months	Statistically significant differences were reported for morale, depression and loneliness. Both groups evidenced a slight decline in morale in loneliness over 12 months, but in both instances, the decline was steeper for the comparison group. The intervention group showed reduced risk of depression after 12 months	Weekly rehearsals for 30 weeks and public perfor-mances	Repeat measures study of a choral singing group. Participants were assigned to an intervention or comparison (usual activity) group	Relatively large sample across three sites. Careful but non-random allocation to intervention and control groups
Cohen.[33] *Journal of Correctional Education*, v60 n1, p52–65/ERIC number: EJ835238/http://www.jstor.org/stable/23282774	Experiment 1 = 20 prisoners aged 22–60.<br>Experiment 2 = 58 including 10 intervention and 10 controls from experiment 1 and male singers from the prison (13) and the community (25)	Friedman Well-Being Scale (FWBS)<br>Pre- and post-performance assessment	In experiment 1 (prisoner choir), interventions and controls both showed significant increases in wellbeing scores ($p = .024$; $p = .003$). In experiment 2, (prisoner + volunteer choir) both groups showed increased wellbeing and there were significant differences between control and experimental groups in four subscales: sociability $p = .003$; joviality ($p = .001$); emotional stability ($p < .001$); and happiness ($p = .016$)	Regular 90-min rehearsals over 9 weeks leading to a perfor-mance	Repeat measures analysis of variance mixed design comparing regular group singing with no singing	Small sample. Single-site study. Self-selected volunteers. Measurements were only collected after a performance, not after rehearsals. Anticipation of leaving prison may have affected the results

(Continued)

Table 1 (Continued)

Characteristics of included quantitative studies.

Authors/title/DOI	Participants recruited (completed)	Wellbeing measures	Wellbeing outcomes reported	Interven-tion and duration	Study design	Quality/bias
Cooke et al.[34,35]/http:// dx. doi. org/10. 1080/1 360786 100371 3190<br>http:// dx.doi. org/10. 1177/1 359105 310368 188	30 male and female (70.2%) participants aged 75–94 years in two residential care settings	Anxiety (RAID), depression and quality of life (GDS)	Participation in the music programme did not significantly affect anxiety, depression and quality of life in older people with dementia. However, GDS scores for a sub group with higher levels of depression at baseline reduced for both groups	40-min sessions three times a week for 8 weeks	Randomised crossover study comparing song singing, music listening and usual care	Small sample. Study population had low levels of anxiety at baseline
Coulton et al.[36]/http://dx. doi.org/10.1192/bjp. bp.113.129908	258 older participants (mean age = 69 years) in a community setting. Females = 214 (83.9)	York SF 12 mental health–related quality of life. Hospital Anxiety and Depression Scale (HAD).<br>Health utility was measured by the EQ5D.21. Assessment at baseline at 3 and 6 months	At 3 months, significant differences between the groups were observed in mental health–related quality of life and anxiety and depression. At 6 months, significant differences were observed in terms of mental health-related quality of life in favour of group singing. The intervention was found to be marginally more cost-effective than usual activities.	Weekly for 14 weeks	A pilot pragmatic individual RCT trial comparing group singing with usual activities	Relatively large sample across five sites within one geographical area where the population is predominantly White British. Findings may not be generalisable to other areas
Field et al.[37]/PMID:92 10776	100 male and female (n = 64) employees aged 22–38 years in a large hospital	STAI<br>POMS<br>Subjective rating scale.<br>Pre- and post assessment	All groups reported decreases in anxiety, depression, fatigue and confusion	10 min	Repeat measures comparing music listening, music relaxation with visual imagery, massage and social support	Hawthorne effect, method of randomisation not stated, limited evidence for immediate effects, no follow-up data

(Continued)

Table 1 (Continued)

Characteristics of included quantitative studies.

Authors/title/DOI	Participants recruited (completed)	Wellbeing measures	Wellbeing outcomes reported	Interven-tion and duration	Study design	Quality/bias
Gold et al.[38]/http://dx.doi.org/10.1177/0306624X13498693	113 male prisoners, mean age 31.38 years	STAI-S<br>Pre-assessment. Post project assessment for intervention group only	Significant improvements recorded in the music group after 2 weeks but no data are available for the control group	2 to 3 sessions of music therapy a week (median 4 sessions)	Pilot RCT assessing group music therapy.<br>Methods included playing in bands, instrumental tuition, recording music, music improvisation, songwriting, and verbal reflections	Sample may not be representative of study population. Low number of intervention sessions reduced the power of the study. Missing data
Guetin et al.[39]/http:// dx.doi.org/10. 1159/0 002290 24	30 residents in nursing home aged 70+ years (22 were female, 8 male.	Anxiety (Hamilton scale) and depression (30-item GDS)	Significant improvements in anxiety and depression in the music therapy group as from week 4 until week 16 and sustained for up to 8 weeks after the discontinuation of sessions	Personal music playlist delivered weekly via headphones for 20 min sessions for 16 weeks	RCT comparing individual music therapy with usual care	Small sample, single-site study. Possible risk of contamination of the comparison group
Gupta and Gupta.[40]/http://dx.doi.org/10.1177/0305735605056144	80 male university students aged 19–24 years	STAI<br>Four-Factor Anxiety Inventory (FFAI)<br>Beck Depression Inventory<br>Baseline assessment and at end	In the music group, a significant ($p < .001$) mean reduction of 2.7 points was recorded on the STAI along with significant improvements in somatic, cognitive, behavioural and affective components of the FFAI ($p < .001$; $p < .1$; $p < .1$; $p < .02$, respectively). No significant changes were reported for the control group	30 min daily for 20 days	RCT comparing listening to slow-paced Indian instrumental music with silence	Generalisability beyond young male sample. Possible risk of contamination of the comparison group

(Continued)

Table 1 (Continued)

Characteristics of included quantitative studies.

Authors/title/DOI	Participants recruited (completed)	Wellbeing measures	Wellbeing outcomes reported	Interven-tion and duration	Study design	Quality/bias
Haslam et al.[41]/http://dx.doi.org/10.1080/13607863.2013.845871	40 older people (mean age = 88.5 years) living in residential facilities including people receiving assisted care	Geriatric Anxiety Inventory<br>The Satisfaction with Life Scale.<br>Baseline assessment and at end.	No significant wellbeing outcomes reported	30-min sessions delivered twice weekly for 6 weeks	RCT comparing three different reminiscence activities: a Story group; a Secular Song Group and a Religious Song Group	Small sample. Risk of contamination. Study excluded participants with significant levels of emotional and mood disturbance
Kim et al.[42]/https://doi.org/10.3349/ymj.2011.52.6.977	18 post-stroke patients in a hospital outpatient department. All were within 6 months of onset and had mini mental status examination (MMSE) scores of over 20	Beck Anxiety Inventory (BAI)<br>Beck Depression Inventory (BDI)<br>Baseline assessment and at end	Anxiety scores decreased slightly in the music group but not in the control group. Depression scores decreased in the music group from a mean of 14.8 (SD = 6.4) to 12.4 (SD = 4.6) after the music therapy, compared with pre: 10.9 (SD = 2.5) and post: 11.1 (SD = 2.5) for the controls. Only the decrease in BDI was significant ($p = .048$)	Twice weekly music therapy sessions for 4 weeks	Intervention and non-random control group study comparing music therapy with no music therapy	Very small sample. Non-random allocation
Koelsch et al.[43]/http://dx.doi.org/10.1525/mp.2010.27.4.307	154 male and female volunteers who considered themselves non-musicians, aged 19–31 years ($m = 24.4$ years)	POMS<br>Pre- and post assessment	Post measures showed significant changes, with depression/anxiety and fatigue decreasing in the music group but not in the control group ($p < .0001$). Vigour increased significantly in the music group ($p < .001$) and decreased in the control group. Irritability did not change in the music group but increased in the control group ($p < .0001$)	Brief session	RCT comparing playing percussion to joyful music with simulated playing to computer generated tones	Exploratory study, good evidence for immediate effects, no follow-up data

(Continued)

Table 1 (Continued)

Characteristics of included quantitative studies.

Authors/title/DOI	Participants recruited (completed)	Wellbeing measures	Wellbeing outcomes reported	Intervention and duration	Study design	Quality/bias
Kreutz.[44] Does singing facilitate social bonding? Music & Medicine https://mmd.iammonline.com/index.php/musmed/article/view/MMD-6-2-9	40 mostly female adults	Ad hoc questionnaire assessing positive and negative feelings	Positive feelings increased significantly after both singing ($p<.01$) and chatting ($p<.01$). Negative feelings decreased significantly after singing ($p<.01$) but not after chatting	Weekly rehearsals for 10 weeks, assessment in weeks 7/8 after 30 min of activity	A naturalistic controlled within-subjects trial singing with a chatting condition	Small sample<br>Limited to a single large piece of classical music: generalisability. Could not consider confounding variables, for example, physical activity
Lee et al.[45]/http://onlinelibrary.wiley.com/doi/10.1111/j.1365-2648.2010.05445.x/abstract	31 adults aged 65 or older in community setting	SF-36<br>The Hong Kong SAR. Assessment at baseline then once a week for 4 weeks	Significant improvements were reported in QoL scores on most of the subscales, including increased vitality, social functioning, role emotional and mental health compared with the control group	Weekly sessions of 30 min duration over 4 weeks	RCT comparing music listening using headphones with rest	Small sample, possible Hawthorne effect, single-site study. Some participants did not enjoy using headphones. Confounding factors may have affected the results
Lord et al.[46]/http://dx.doi.org/10.1186/1471-2466-12-69	33 (24) patients with COPD attending respiratory clinics in a UK NHS Trust	SF-36<br>Baseline assessment and at end.	Both control and intervention showed significant improvements in the mental component score of the SF-36: singing + 9.3 (25.3) versus Film + 4.3(9.0) ($p=.41$). There was a significant difference between the response of the physical component score favouring the singing group + 12.9 (19.0) versus .25 (11.9) ($p=.02$)	Weekly classes for 8 weeks	RCT comparing singing classes with film club	Small sample. Singing group met twice weekly while the film group met weekly

(Continued)

Table 1 (Continued)

Characteristics of included quantitative studies.

Authors/title/DOI	Participants recruited (completed)	Wellbeing measures	Wellbeing outcomes reported	Interven-tion and duration	Study design	Quality/bias
Perkins and Williamon.[47]/ http://dx.doi.org/10.1177/ 0305735613483668	98 mostly female older adults (mean age = 67.87 years)	7-item short WEMWBS<br>52-item Health Promoting Lifestyle Profile II (HPLPII) Qualitative interviews with 21 music learners<br>Baseline assessment and at end	Wellbeing scores improved significantly for all groups. Qualitative analysis revealed themes including pleasure, social interactions, musical engagement, musical ambition, ability and self-satisfaction	10-week programme	Mixed methods study comparing instrumental lessons with no lessons	Non-random, convenience sample
Särkämö et al.[48]/http:// dx. doi. org/10. 1093/g eront/g nt100	89 People with dementia and carer dyads recruited from 5 different day activity centres and inpatient centres	Mood and QOL (Cornell-Brown Scale for Quality of Life and QOLAD scales.	Singing and listening to familiar songs, provided by the caregivers of PWDs can be beneficial. Both singing and music listening improved mood. Singing improved emotional wellbeing of their family members, whereas music listening was found to improve the QOL of the PWDs.	Weekly 1.5 h music sessions for 10-week. In addition, the intervention included regular musical exercises at home	RCT comparing singing, music listening and usual care.	Dementia diagnosis not specified. Relatively short intervention time.<br>Study population all scored highly at baseline on a scale for rating daily listening to music and approximately 50% of each group had a history of music and singing since childhood.
Solé et al.[49]/http://dx.doi. org/10.1093/jmt/47.3.264	83 older participants (mean age = 72.6 years) in a community setting. Females = 69<br>(83.13%)	Ad hoc QoL questionnaire.<br>Baseline assessment and at end	No significant changes recorded although participants perceived improvements in some aspects	1 academic year (October – June)	Evaluation of singing, music appreciation class or music therapy	Small groups, non-random assignment, Hawthorne effect and risk of contamination

(Continued)

Table 1 (Continued)

Characteristics of included quantitative studies.

**Authors/title/DOI**	**Participants recruited (completed)**	**Wellbeing measures**	**Wellbeing outcomes reported**	**Interven-tion and duration**	**Study design**	**Quality/bias**
Sun and Buys.[50]/DOI: 10.1515/ijamh.2012.040	113 (72) adults with chronic conditions recruited from community health services	Singing-related QoL.<br>Baseline assessment and at end	When the singing group was compared with usual care alone, statistically significant improvements were seen after 12 months, with increases in singing-related mental and physical health aspects of QoL and positive affect ($p<.001$ and $p<.001$)	Weekly 2h choir rehearsals for 12 months	Intervention and control group study comparing singing with usual care	Self-selected, non-random allocation. Smaller control group ($n=27$)
Sun et al.[51] http://dx.doi.org/10.1080/14623730.2013.842337	750 adults aged 51–85 (mean 61.9), the majority of whom presented with risk factors for chronic diseases.	Self-completed health benefit questionnaire<br>The General Health Questionnaire (GHQ30) The Resilience scale (Fribor et al. 2003)<br>Baseline assessment at end	The Participants in TC, dancing and singing groups showed significant differences in all health benefits and resilience outcome data in comparison with the control group. There were significant differences in depression scores: all meditative groups had a lower incidence of depression (2.5%–7.5%) than the control group (10.1%), and the TC and dancing groups had the lowest depression rate among the four intervention groups	15-month programme offering tai chi, dancing, playing a musical instrument or singing	Case-controlled design. After they had chosen their preferred intervention, each participant was matched with regard to their medical condition to a participant in the control group. Controls joined in educational activities	Non-random allocation to groups. There was no combined exercise group so the effects of singing and TC or dancing cannot be examined
Valentine and Evans.[52] /http://dx.doi.org/10.1348/000711201160849	33 male and female university students (mean age = 21.2 years)	UWIST model adjective checklist (UMACL)<br>Pre- and post assessment	Improved mood was recorded for all groups, with the strongest changes for swimming	30 min	Non-random study comparing solo singing, group singing and swimming	Small sample, single-site, non-random allocation. Short intervention time
Warth et al.[53]/http://dx.doi.org/10.3238/arztebl.2015.0788	84 (68) Cancer patients (mean age = 63 years, 71.4% were female) receiving palliative care in hospital.	Visual Analog Scales (VAS) recording self-rated relaxation and wellbeing.<br>Pre- and post assessment	Significantly greater increases in the music therapy group compared to the control group ($p<.001$ and $p=.013$, respectively)	Two 30-min music therapy sessions	RCT comparing music therapy delivered by a therapist following a protocol with relaxation	Small, single-site study. Short intervention time

(Continued)

Table 1 (Continued)

Characteristics of included quantitative studies.

Authors/title/DOI	Participants recruited (completed)	Wellbeing measures	Wellbeing outcomes reported	Interven-tion and duration	Study design	Quality/bias
Wlodarczyk.[54]/http://dx.doi.org/10.1093/jmt/44.2.113	10 hospice patients	Spirituality questionnaire. Pre- and post assessment	Significant improvements in the intervention periods compared with the control periods ($N=10$, $d=1$, $p=.01$)	30-min music therapy session	Patients acted as own controls, random allocation to ABAB or BABA conditions in order to compare music therapy (including singing and worship) with no music therapy	Very small sample, short intervention time
Wu.[55]/http://doi.org/10.2117/psysoc.2002.104	28 (24) Male and female university students diagnosed with major or minor depression, anxiety and low self-esteem	Zung Self-Rating Anxiety Scale (SAS). Zung Self-Rating Depression Scale (SDS) Self-esteem scale (SES) (Rosenberg 1965). Baseline assessment and at end	At follow up, there were significant differences in anxiety (and in anxiety and depression at 2 months follow-up) were found for the music therapy group relative to the control group ($p<.01$ in both instances)	Weekly 2 h sessions for 10 weeks	RCT comparing music therapy sessions with no music therapy	Very small sample in a single setting. Music activities not specified

RCT: randomised controlled trial; PE: physical education.
